# Short and long-arm fiberglass cast immobilization for displaced distal forearm fractures in children: a randomized controlled trial

**DOI:** 10.1007/s00264-020-04800-w

**Published:** 2020-09-17

**Authors:** Michelle Seiler, Peter Heinz, Alessia Callegari, Thomas Dreher, Georg Staubli, Christoph Aufdenblatten

**Affiliations:** 1 Pediatric Emergency Department, University Children’s Hospital Zurich, Steinwiesstrasse 75, 8032 Zurich, Switzerland; 2 Children’s Research Center, University Children’s Hospital Zurich, University of Zurich, Zurich, Switzerland; 3 Department of Pediatric Surgery, Division of Trauma and Orthopedic Surgery, University Children’s Hospital Zurich, Zurich, Switzerland

**Keywords:** Short-arm cast, Long-arm cast, Fiberglass cast, Distal forearm fracture, Paediatrics, Closed reduction

## Abstract

**Purpose:**

The aim of this study was to investigate whether short-arm fiberglass cast (SAC) immobilization provides fracture stabilization comparable to that of long-arm cast (LAC) treatment of displaced distal forearm fractures after closed reduction in paediatric patients.

**Methods:**

A prospective, randomized, controlled trial of children aged four to 16 years (mean 9.9 years) was designed with a sample of 120 children, whose size was set a priori, with 60 treated with SAC and 60 with LAC. The primary outcome was fracture stability and rate of loss of reduction. The secondary outcome analysis evaluated duration of analgesic therapy, restriction in activities of daily life, and the duration until patients regained normal range of motion in the elbow.

**Results:**

No statistically significant differences were found between the two groups in loss of reduction or duration of analgesic therapy. In contrast, the duration until normal range of motion in the elbow was regained was significantly longer in the LAC group (median 4.5 days, *P* < 0.001). Restriction in activities of daily life did not differ significantly between the two groups except for the item “help needed with showering in the first days after trauma” (SAC 60%, LAC 87%, *P* = 0.001).

**Conclusion:**

Fracture immobilization with short-arm fiberglass cast in reduced distal forearm fractures is not inferior to long-arm casts in children four years and older, excluding completely displaced fractures. Furthermore, short-arm casting reduces the need for assistance during showering.

**Trial registration:**

NCT03297047, September 29, 2017

Distal forearm fractures (DFFs) are the most common fractures in childhood, accounting for 40% of all fractures in children [1]. The gold-standard imaging technique to determine the fracture type, the degree of fracture angulation, and fracture displacement is projectional radiography in two planes [2]. Non-operative management with closed reduction and splinting of displaced fractures is the treatment of choice when closed reduction leads to a stable reduction, whereas surgical treatment is generally necessary for unstable, open, and intra-articular fractures [1]. Unfortunately, non-operative treatment of previously reduced fractures contains the possibility of a loss of reduction (LOR), which occurs at a rate of 12–34% [1, 2].

Factors associated with LOR include poor casting technique, initial complete displacement, and failure to achieve perfect anatomical reduction after closed reduction [1, 3, 4]. Several casting indices have been published that assess the quality of casting technique, and they focus mainly on the molding. However, their clinical value has been questioned and they seem to be unsuitable for fiberglass (FG) casts, because FG casts leave wider gaps between the cast and skin [1, 3, 5]. Completely displaced fractures are associated with a risk for LOR of up to 25% due to the instability caused by rupture of the periosteum and distinct swelling caused by severe soft tissue injury [1, 3]. An inadequate initial fracture reduction increases the risk for LOR fivefold [1, 3]. Therefore, standard follow-up regimes must include radiographic monitoring with posteroanterior (PA) and lateral radiographs to ensure proper fracture healing in an acceptable position.

The remodeling potential of distal metaphyseal forearm fractures in children is high, and angulation deformities between 15 and 40° may be tolerated, depending on the age and the distance of the fracture from the physis [1, 2, 5]. However, a 20° angulation in the sagittal plane and a 10° angulation in the frontal plane are generally recommended as the upper limit for children with open physes to avoid a prolonged period of remodeling during which pronation and supination are restricted [5, 6].

Fracture immobilization in children is usually managed with upper-arm plaster of Paris casts or FG casts. Long-arm casts (LACs) are mostly recommended for reduced DFF treatment because they prevent pronation and supination movement [7–11]. However, short-arm casts (SACs) are easier to apply, result in fewer restrictions for activities of daily life (ADL), and cause less elbow stiffness [12]. Studies comparing above- and below-elbow plaster of Paris casts concluded that SACs seemed to be as effective as LACs in stabilizing fractures [7–11].

However, to the authors’ best knowledge, no studies have investigated whether the same applies to FG casts.

The aim of this study was to assess whether fracture immobilization after closed reduction with short-arm FG casts is as effective in displaced forearm fracture stabilization as long-arm FG casts in children age four years and over.

## Materials and methods

A prospective, randomized trial was performed. After approval by the local ethics board and registration on ClinicalTrials.gov (NCT03297047), enrolments were made at the authors’ institution from the emergency department (ED) between October 2017 and July 2019.

Patients aged four to 16 years with displaced metaphyseal or epiphyseal DFFs requiring closed reduction were eligible for participation in this study. Younger patients were not included due to the risk of the forearm cast slipping off. To increase the validity of this study, completely displaced fractures were excluded because different opinions exist whether such fractures benefit from osteosynthesis after reduction or not [13, 14]. Other exclusion criteria were open fractures, pathologic fractures, intra-articular fractures, and fully or partially closed physes in adolescents. A metaphyseal fracture was defined as a fracture within a square over the epiphyseal plate of both forearm bones on a PA radiograph [15]. Written informed consent was obtained from all parents of all children and from patients 14 years and older, while verbal consent was obtained from all children between 11 and 14 years.

The patients were allocated randomly into the two groups (SAC, LAC). After confirming study participation, patients drew a sealed envelope enclosing a numbered card, which assigned them to one group or other. After choosing the envelope, neither the ED staff nor the parents and their children were blinded for the treatment groups. However, the data were analyzed under blinded conditions.

Fractures were reduced by experienced consultants at the ED, and the casts were applied by specially trained nurses. Indication for the reduction was a fracture angle > 10° in the frontal plane or > 20° in the sagittal plane in patients younger than 11 years, and fracture angle > 10° in either plane in older patients. Inhalative nitrous oxide 70% was administered for analgosedation in all cases. Standardized radiographs were obtained after reduction to determine correct alignment.

After reduction, a circular Scotchcast® (3M, Ruschlikon, Switzerland) was applied. Cast structure was a soft-Scotch cast with extension either below the elbow or above the elbow combined with a rigid fiberglass splint on the forearm’s (SAC) or whole arm’s dorsal (LAC) and on the forearm’s volar side, followed by a final soft-Scotch layer. At the authors’ institution, fiberglass splint is preferred to plaster of Paris casts due to its light weight, tolerance of water, and variety of available colours, which are attractive for children.

Monitoring of the healing of the reduced and splinted DFFs followed a standardized protocol with regular follow-up examinations at the pediatric orthopedic outpatients’ clinic on days five, ten and 28 and week seven.

To answer the primary research question of this study, PA, and lateral radiographs at initial presentation, after reduction, and at days five, ten and 28 were assessed using an established standardized method to ensure precision and accuracy; one line was drawn along the midshaft of the diaphysis and the other line along the midshaft of the metaphysis/epiphysis, and the angle between these two lines was defined as fracture angulation [5].

Furthermore, five demographic parameters were analyzed: type of fracture (metaphyseal, Salter-Harris type I, or Salter-Harris type II), isolated fracture of the radius or of the entire forearm (radius and ulna), whether the dominant arm was injured, the initial fracture angulation, and residual fracture angulation after reduction and at the first three follow-up visits.

LOR was defined as angular deviation in the lateral view > 20° in patients younger than 11 years and > 10° in older patients. A secondary fracture displacement meant early study termination if the treating orthopedist decided that the fracture required either another closed reduction with fracture stabilization in a LAC or another reduction and fracture stabilization with osteosynthesis. All study charts were regularly reviewed by one of the authors, and all radiographic measurements were performed by a trained study nurse. One of the authors performed random tests for quality assurance, and all measurements were within ± 5°.

The secondary outcome was the assessment of analgesic need and restriction in ADL and the duration needed until unrestricted movement of the elbow was possible following cast removal in different age subgroups. At the first three follow-up visits, children and parents were asked how many days analgesic medication (paracetamol and non-steroidal anti-inflammatory) was required and whether the child needed help in ADL (yes/no questions). At the final visit 7 weeks after trauma, children and parents were asked about the duration needed for the children to use their elbow unrestrictedly in ADL. Additionally, the range of motion (ROM) in the elbow was measured (flexion and extension).

### Statistical analysis

For the a priori sample size calculation, we assumed that a risk difference of up to 5% in secondary displacement rate was clinically irrelevant. Given this criterion and assuming the risk difference of a SAC to be − 10%, we can establish non-inferiority with a power of 90% and an alpha error 0.05 with 60 patients in each group.

The rate of redisplacements was compared with the odds ratio (OR) and 95% confidence interval (95% CI). Normally distributed data were presented as means and standard deviation (SD), data of other distributions as medians and interquartile range (IQR). Differences between groups were analyzed using the chi-square test for categorical data and a two-sample *t* test for continuous data. Levene’s test for equality of variance was used to test for homogeneity of variance. Correlation analysis used Pearson correlation. To compare continuous variables of angulation change in degrees between time points, an analysis of variance (ANOVA) for repeated measures was used. Sphericity was tested with Mauchly’s tests. For all tests, values of *P* < 0.05 were considered statistically significant. Statistical analyses were performed with SPSS Statistics V.24 (SPSS Inc., IBM Company, Chicago, IL, USA).

## Results

**Fig. 1:**
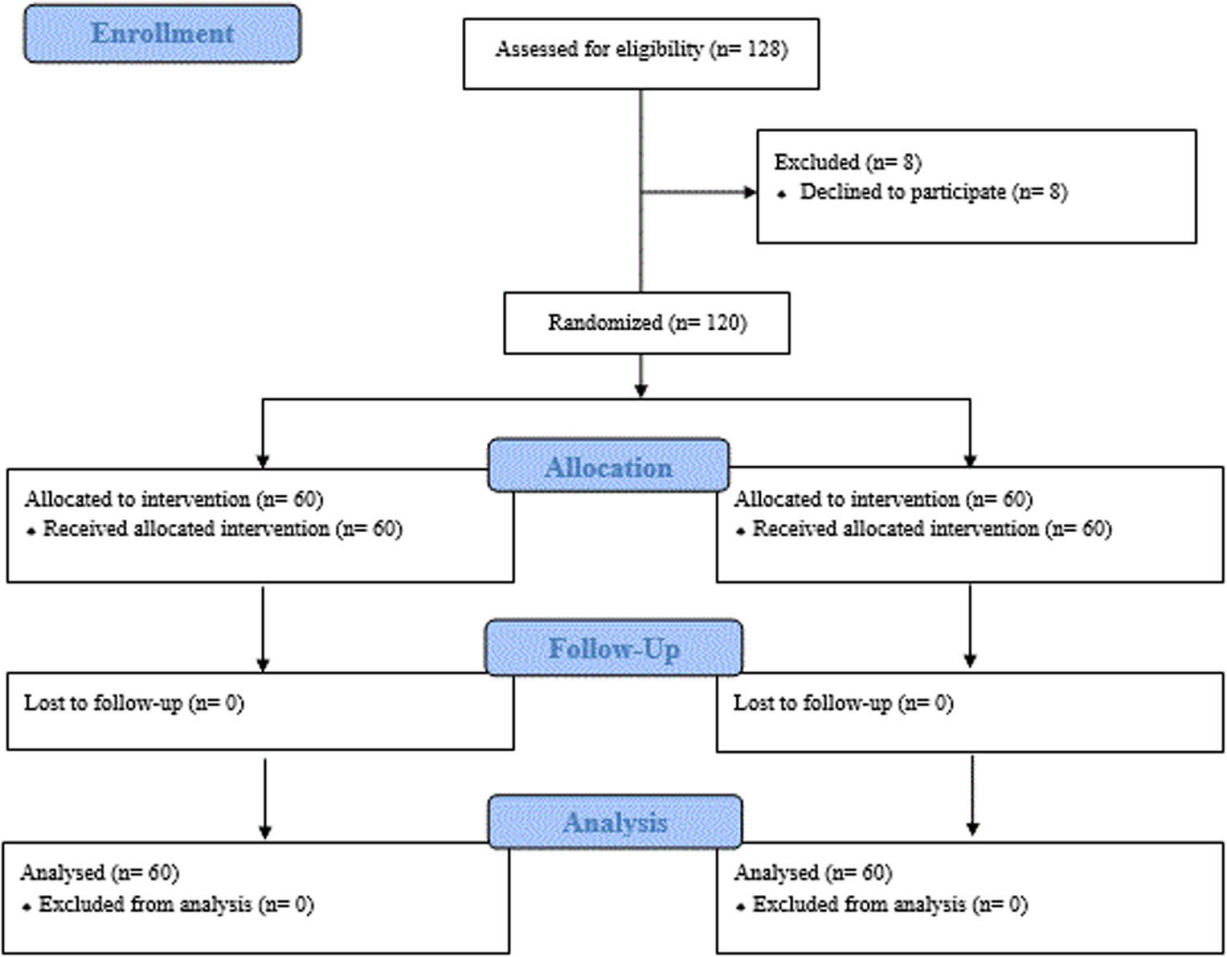
CONSORT (Consolidated Standards of Reporting Trials) diagram, showing participant flow, patients screened, excluded, randomized, followed, and analyzed in this study

During the two year study period, 128 children met the enrollment criteria and were invited to participate, but 8 parents refused to participate. Thus, 120 children with displaced DFFs were enrolled, of whom 60 were treated with a SAC and 60 with a LAC (Fig. 1). The mean age was 9.9 years (range 4–16 years), and the two groups did not differ with regard to demographics, fracture specifications, or the dominant hand side (see Table 1).

**Table 1:** Characteristics of study patients and fracture specifications for the short-arm cast (SAC) and long-arm cast (LAC)

	SAC group (*n* = 60)	LAC group (*n* = 60)	Both groups (*n* = 120)	*P*
Age	9.9 ± 3.2	9.9 ± 2.9	9.9 ± 3.0	0.83
4–7 years	17	14	31	
8–11 years	20	26	46	
12–16 years	23	20	43	
Sex (male)	38 (63.3)	32 (53.3)	70 (58.3)	0.27
Fractures	60 (50)	60 (50)	120 (100)	
Displaced metaphyseal fracture	52	49	101	0.45
Displaced Salter-Harris II fracture	7	10	17	0.43
Displaced Salter-Harris I fracture	1	1	2	1.0
Fracture of radius and ulna	29 (48.3)	32 (53.3)	61 (50.8)	0.58
Isolated fracture of the radius	31 (51.7)	28 (46.7)	59 (49.2)	0.58
Fracture on dominant hand site	27 (45)	23 (38.3)	50 (41.7)	0.46

Data presented as mean ± SD/*n*/*n* (%)

### Primary outcome analysis

**Fig. 2:**
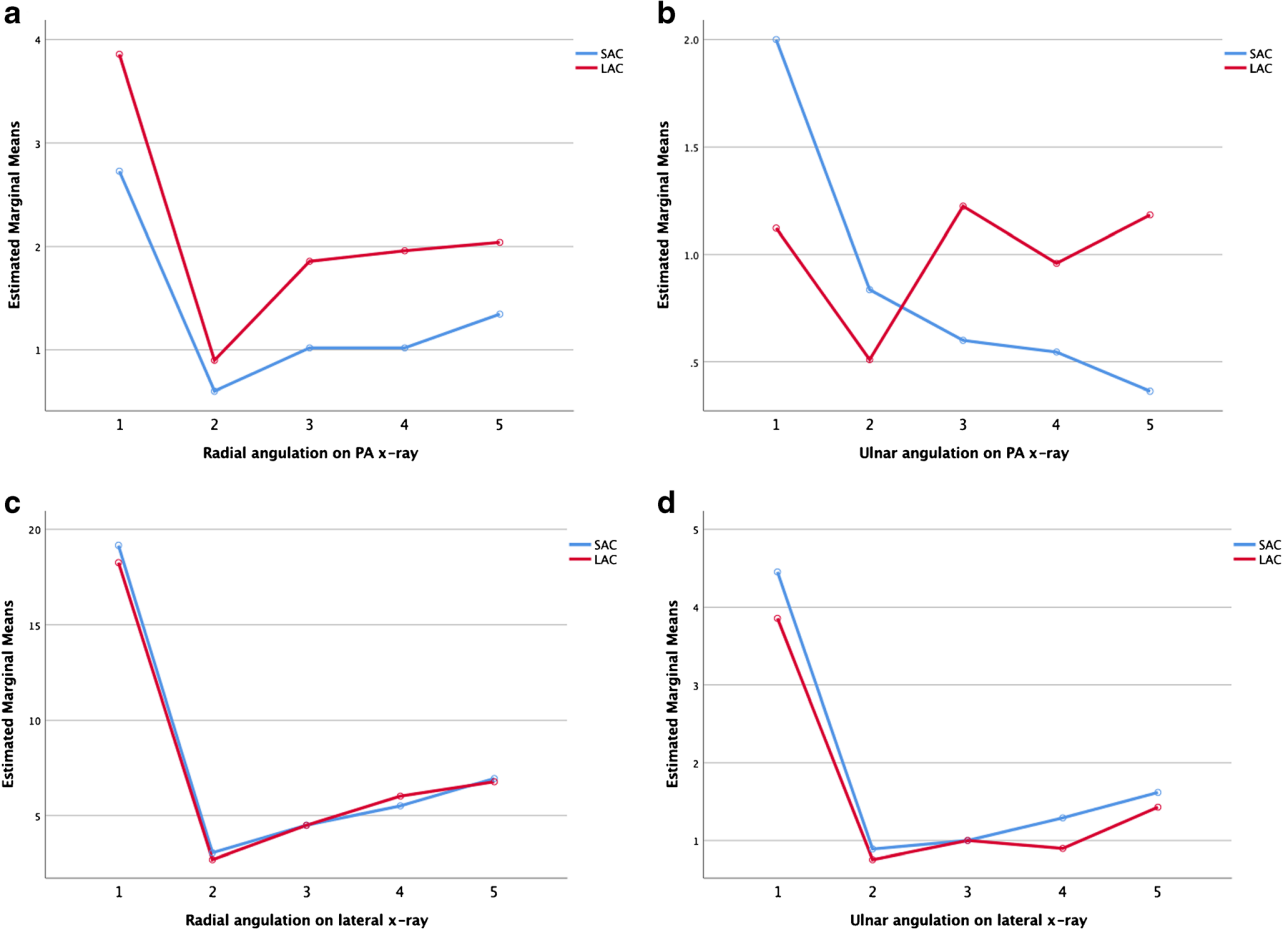
Analysis of variance (ANOVA) of fracture angulation in degrees between time points (on days 0, 5, 10, and 28). SAC, short-arm cast; LAC, long-arm cast; PA, posteroanterior. **a** radial angulation on PA X-ray (*P* = 0.65); **b** ulnar angulation on PA X-ray (*P* = 0.01); **c** radial angulation on lateral X-ray (*P* = 0.7); **d** ulnar angulation on lateral X-ray (*P* = 0.8)

The two patient groups showed no statistically relevant difference in fracture angulation values either at pre-treatment or during follow-up, as shown in Table 2. ANOVA analyses found no interaction between cast type and displacement over time, except for the ulnar PA plane (*P* = 0.01; Fig. 2).

**Table 2:** Radiographic values for short-arm cast (SAC) and long-arm cast (LAC)

	SAC group (*n* = 60)	LAC group (*n* = 60)	*P*
Pre-treatment			
Radial angulation on PA X-ray	1.0 (4.7)	2.0 (5)	0.58
Ulnar angulation on PA X-ray	0 (0.7)	0 (0)	0.51
Radial angulation on lateral X-ray	18 (8.5)	17.5 (8.5)	0.38
Ulnar angulation on lateral X-ray	0 (4.7)	0 (6.7)	1.0
Post-reduction			
Radial angulation on PA X-ray	0 (0)	0 (0)	0.82
Ulnar angulation on PA X-ray	0 (0)	0 (0)	0.46
Radial angulation on lateral X-ray	3 (5)	3 (4.7)	0.58
Ulnar angulation on lateral X-ray	0 (0)	0 (0)	0.82
5-day visit			
Radial angulation on PA X-ray	0 (2)	0 (3)	0.06
Ulnar angulation on PA X-ray	0 (0)	0 (0)	0.63
Radial angulation on lateral X-ray	4.5 (5.7)	4 (5)	1.0
Ulnar angulation on lateral X-ray	0 (0)	0 (0)	1.0
10-day visit			
Radial angulation on PA X-ray	0 (2)	0 (3)	0.28
Ulnar angulation on PA X-ray	0 (0)	0 (0)	0.67
Radial angulation on lateral X-ray	5 (6.5)	6 (6.5)	0.73
Ulnar angulation on lateral X-ray	0 (2)	0 (0)	0.48
At cast removal			
Radial angulation on PA X-ray	0 (2)	0 (3)	0.93
Ulnar angulation on PA X-ray	0 (0)	0 (0)	0.22
Radial angulation on lateral X-ray	6 (8.2)	6.5 (4.2)	0.59
Ulnar angulation on lateral X-ray	0 (3.2)	0 (3)	0.98

Data presented as median (IQR); angulation in degrees

LOR was seen in 25 cases (21%), of whom 10 were treated with a SAC (17% within the group) and 15 were treated with a LAC (25% within the group) (*P* = 0.26, OR 1.67, 95% CI 0.68–4.08). The mean LOR angulation in the SAC group was 16.7° (SD 6.08) and 15.3° in the LAC group (SD 4.67). Remanipulations were performed in 8 cases (6.7%), of whom two were treated with a SAC (3.3% within the group) and six were treated with a LAC (10% within the group) (*P* = 0.14).

### Secondary outcome analysis

No differences were found between the groups in days of analgesic therapy or limitations in ADL (showering, getting dressed, eating, going to the lavatory, brushing the teeth) except with the item “help with showering” (Table 3). Taking a shower was the main limitation for patients with a LAC: in fact, 87% (52/60) of those with a LAC and 60% (36/60) of those with a SAC required help in the first days after trauma (*P* = 0.001). In days 11–28 after trauma, 72% (39/60) of the patients with a LAC needed help taking a shower (*P* = 0.02), in comparison with 50% (29/60) of the SAC group.

**Table 3:** Assessment of analgesic medication and restriction in activities of daily life

	SAC group (*n* = 60)	LAC group (*n* = 60)	*P*
Days of analgesic medication*	2 (3)	3 (3)	0.10
Activities of daily life^§^			
First 5 days			
Help with showering	36 (60)	52 (86.7)	*0.001*
Help with getting dressed	42 (70)	42 (70)	1.0
Help with eating	23 (38.3)	18 (30)	0.33
Help with going to the lavatory	15 (25)	12 (20)	0.66
Help with brushing the teeth	13 (21.7)	11 (18.3)	0.82
Days 6–10			
Help with showering	35 (59.3)	40 (74.1)	0.11
Help with getting dressed	28 (47.5)	30 (55.6)	0.45
Help with eating	12 (20.3)	12 (22.2)	0.82
Help with going to the lavatory	10 (16.9)	8 (14.8)	0.80
Help with brushing the teeth	12 (20.3)	8 (14.8)	0.47
Days 11–28			
Help with showering	29 (50)	39 (72.2)	*0.02*
Help with getting dressed	17 (29.3)	25 (46.3)	0.08
Help with eating	7 (12.1)	6 (11.1)	1.0
Help with brushing the teeth	3 (5.2)	2 (3.7)	1.0
Help with going to the lavatory	5 (8.6)	8 (14.8)	0.38
Time to regain normal motion of the elbow*	0	4.5 (6)	*< 0.001*
Age group 4–7 years	0	2 (3)	*< 0.001*
Age group 8–11 years	0	4.5 (6)	*< 0.001*
Age group 12–16 years	0	7 (8)	*< 0.001*

*Data presented as median (IQR) in days

^§^Data presented as *n*/*n* (%)

Italic data represents the significance for *p* < 0.05 with chi square for caregorical and two *t* test for continuous data

**Fig. 3:**
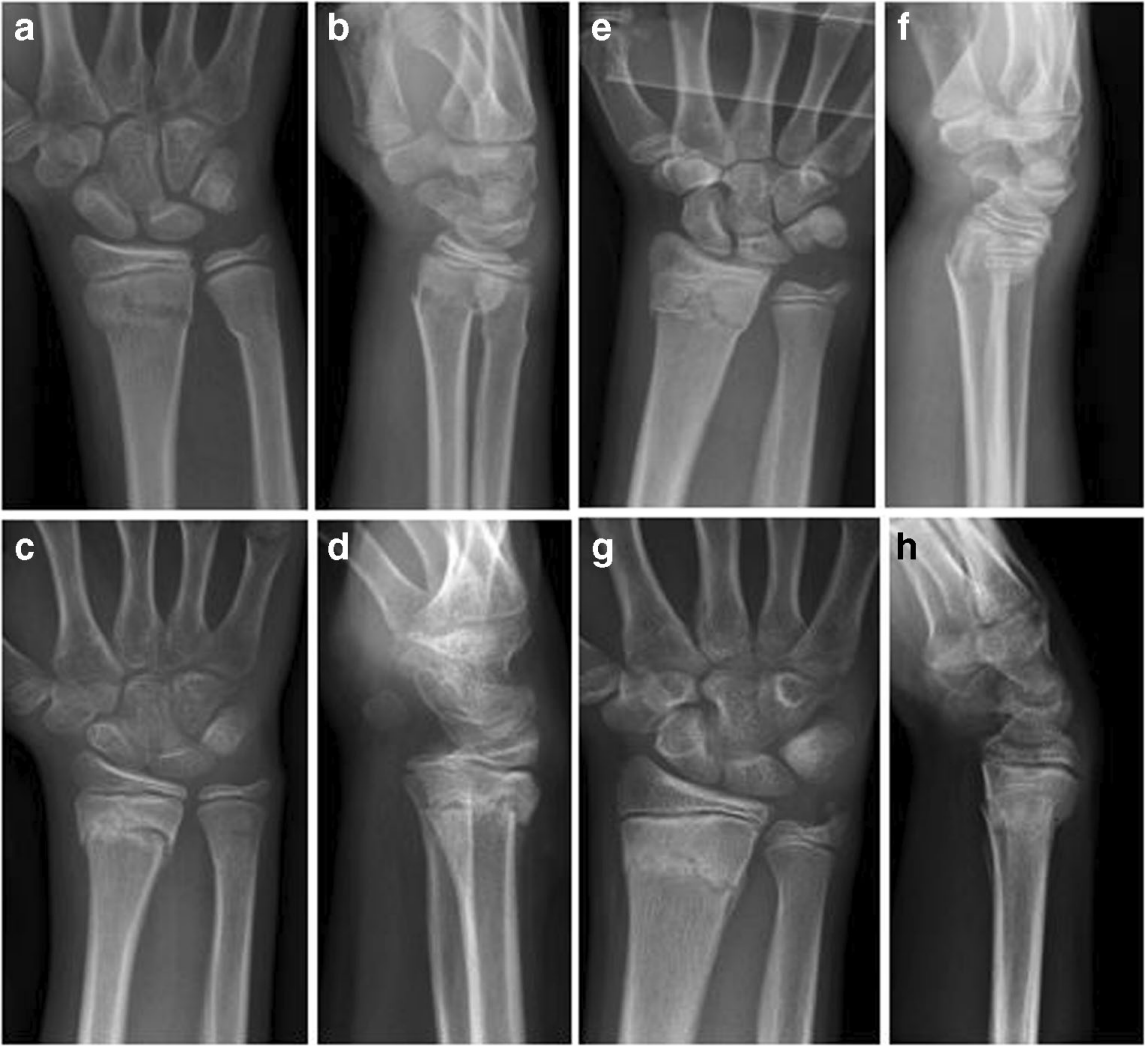
Two cases of metaphyseal fractures with posteroanterior and lateral radiographs at initial presentation before closed reduction and after 28 days. **a**–**d** show the X-rays of a 10-year-old female patient treated with a SAC. The initial X-rays before treatment (**a**, **b**) and the X-rays after 4 weeks (**c**, **d**) are shown. Unrestricted movement of the elbow was possible at the time of cast removal with a normal ROM of the elbow 3 weeks later (extension/flexion 0/0/150°). **e**–**g** A 12-year-old male patient treated with a LAC. **e** and **f** show the fracture before closed reduction, **g** and **h** after 4 weeks of LAC treatment. Restricted range of movement of the elbow for 9 days after cast removal. Three weeks later, ROM of the elbow was 0/0/140°

**Fig. 4:**
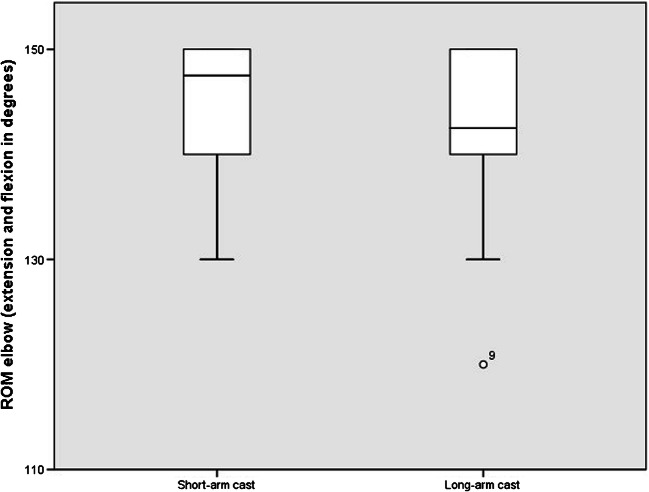
Graphical display of ROM of elbow flexion and extension 3 weeks after cast removal, comparing short-arm cast (SAC) versus long-arm cast (LAC)

Self-reported unrestricted motion of the elbow was reached 4.5 days after removal of the cast in the LAC group, whereas the SAC group reported no restriction (Table 3). At week seven, elbow flexion and extension were unrestricted in all patients except one with LAC treatment; however, he recovered completely within one week without any further treatment. Figure 3 shows the radiologic imaging of two similar cases with comparable fractures, one treated with a SAC, one treated with a LAC, respectively. ROMs of the elbow after treatment are displayed in a graphic (Fig. 4).

**Fig. 5:**
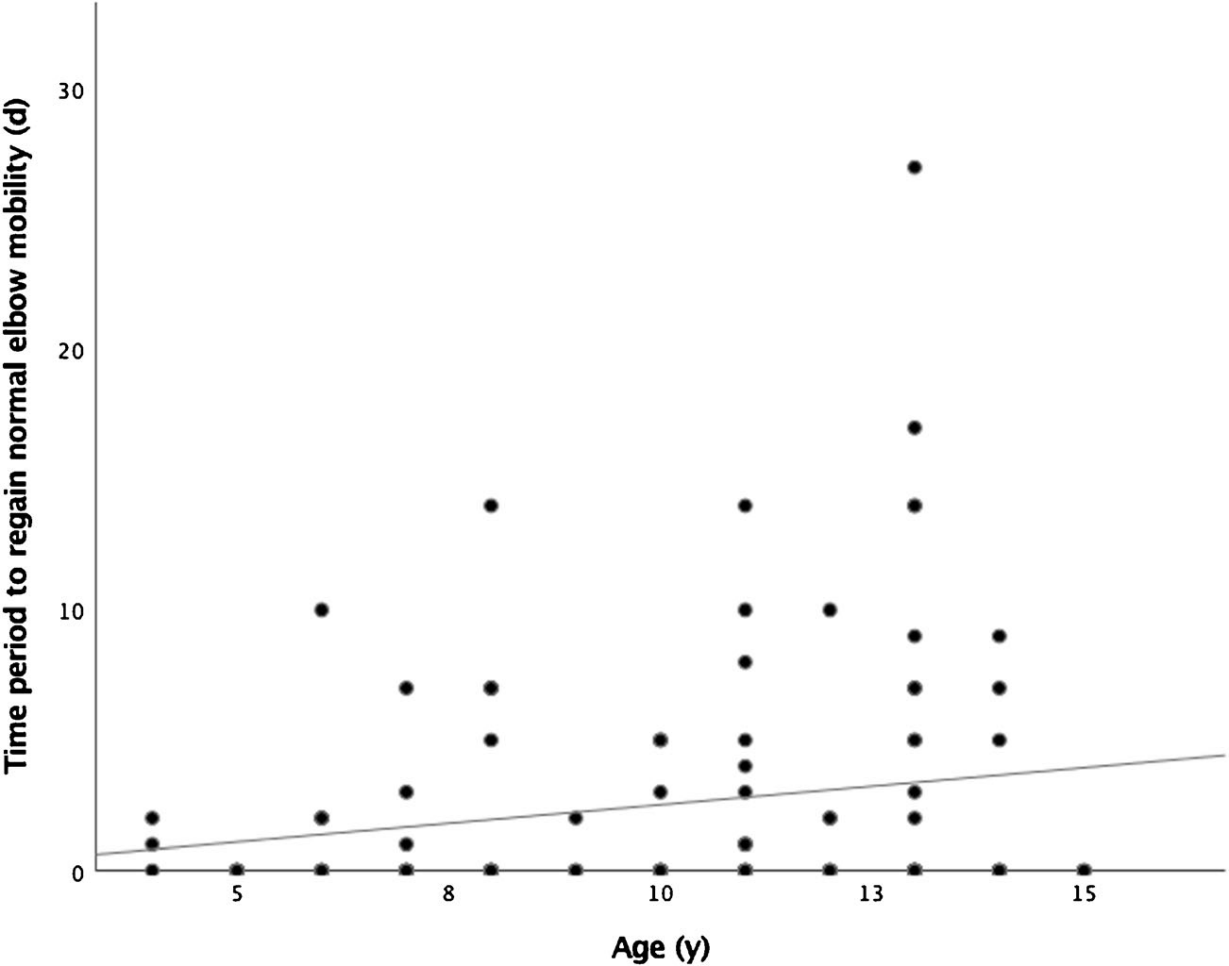
Time period to regain normal elbow mobility, (d, days; y, years). Graph illustrating Pearson correlation for the time needed in different age groups (*r* = 0.19)

The age subgroups in the LAC group took progressively longer from four to seven years to 12–16 years to regain normal mobility of the forearm (Table 4, Pearson correlation is shown in Fig. 5).

**Table 4:** Treatment success and loss of reduction (LOR)

	Success (*n* = 95)	LOR (*n* = 25)	*P*
Age	9.6 ± 3.0	11.2 ± 2.7	*0.02*
Type of fracture			
Displaced metaphyseal fracture	76 (80)	25 (100)	*0.05*
Displaced Salter-Harris II fracture	17 (17.9)	–	
Displaced Salter-Harris I fracture	2 (2.1)	–	
Both bone fracture	47 (49.5)	14 (56)	0.56
Fracture on dominant hand site	43 (45.3)	7 (28)	0.12

Data presented as mean ± SD/*n* (%)

### Other outcomes

Independent of the type of casting, LOR occurred more often in older children, in those with metaphyseal fractures, and with incomplete anatomical fracture reduction for radial angulation on lateral radiographs (comparing Tables 4 and 5). In children with LOR, radial angulation in the sagittal plane was statistically significantly higher in all follow-up visits than in children whose treatment was successful (Table 5).

**Table 5:** Radiographic values for treatment success and loss of reduction (LOR)

	Success (*n* = 95)	LOR (*n* = 25)	*P*
Pre-treatment			
Radial angulation on PA X-ray	2 (5)	2 (4)	0.73
Ulnar angulation on PA X-ray	0 (0)	0 (4)	0.96
Radial angulation on lateral X-ray	18 (8)	16 (6)	0.76
Ulnar angulation on lateral X-ray	0 (7)	0 (6)	0.76
Post-reduction			
Radial angulation on PA X-ray	0 (0)	0 (2)	0.26
Ulnar angulation on PA X-ray	0 (0)	0 (1)	0.84
Radial angulation on lateral X-ray	2 (4)	4 (6)	*0.006*
Ulnar angulation on lateral X-ray	0 (0)	0 (1)	0.62
5-day visit			
Radial angulation on PA X-ray	0 (2)	0 (7)	0.60
Ulnar angulation on PA X-ray	0 (0)	0 (4)	0.94
Radial angulation on lateral X-ray	4 (4)	7 (6)	*0.005*
Ulnar angulation on lateral X-ray	0 (0)	0 (3)	0.95
10-day visit			
Radial angulation on PA X-ray	0 (2)	2 (7)	0.10
Ulnar angulation on PA X-ray	0 (0)	0 (4)	0.44
Radial angulation on lateral X-ray	4 (5)	11 (6)	*< 0.001*
Ulnar angulation on lateral X-ray	0 (0)	0 (1)	0.68
At cast removal			
Radial angulation on PA X-ray	0 (2)	2 (6)	0.18
Ulnar angulation on PA X-ray	0 (0)	0 (0)	0.95
Radial angulation on lateral X-ray	6 (6)	9 (8)	0.17
Ulnar angulation on lateral X-ray	0 (2)	0 (5)	0.71

Data presented as median (IQR); angulation in degrees

## Discussion

Our randomized controlled study demonstrated that fracture immobilization of reduced DFF with FG short-arm casting is as effective as the treatment with long-arm casting, excluding completely displaced fractures.

Fracture stabilization in this study was high, with a LOR rate of 21% with no significant differences between the two cast groups. These results corroborate the conclusions of previous studies [7–11]. Gold-standard immobilization for such fractures is a molded plaster of Paris cast, which has a reported LOR rate between 12% and 34% [1, 2]. So, since our study results for LOR do not differ from previous studies, FG casting does not seem inferior to the plaster of Paris casting. Additionally, the duration of analgesic medication required in our study was comparable with previously published data, which found that the mean duration for pain medication requirement was 3.2 days [16]. Therefore, FG SAC is not inferior to FG LAC and can be used as an alternative to plaster of Paris casts for reduced DFF.

Assessment of the limitations in ADL showed that showering with a cast was the most difficult task and required support most often, especially in those with a LAC. This difference did not disappear until the removal of the cast after 4 weeks. Concerning other ADL, in contrast to the study performed by Webb et al., we did not find group differences in help required with dressing, eating, and going to the lavatory [9]. This indicates that FG casts potentially cause fewer limitations in ADL; however, the study design does not enable a conclusive answer to this question.

Recovery after removal of the cast was significantly quicker in the SAC group than in the LAC group. The time needed to regain normal motion of the broken arm was considerably shorter than results published so far, which used the same methodology with self-reported assessment [9]. The main difference from our study was the use of plaster of Paris casts instead of FG casts and a longer fracture immobilization in a cast (40 days compared with 28 days in our study) [9]. The shorter duration of immobilization in our study was congruent with recommendations in literature that advise immobilization for only four weeks in children [12]. Our data show that time span until the normal motion of the elbow was three times as long in children aged 12–16 years as in children aged four to seven years in the LAC group. Spencer et al. found similar results and concluded age to be a significant factor in the recovery of elbow motion after cast removal [17]. Older children might be more cautious or fearful of starting to use the elbow after cast removal, whereas the younger children start using their healed arms without further reflection.

LOR was similar in both groups regardless of the length of the cast, but it occurred more often in older children with higher post-reduction angulation of the radius in the sagittal plane. No differences were found in primary fracture angulation and fracture type between the children with successful treatment and those in whom LOR occurred. In contrast to previous studies [5], no association was found between LOR and either an isolated distal radial fracture or a combined fracture of the radius and ulna. Isolated distal radius fractures even have an increased risk for LOR due to the difficulty of obtaining sufficient initial fracture reduction and maintaining fracture alignment [5]. In our analysis, all fractures with LOR were metaphyseal DFFs, which represented the most common type of fracture in our study (84%). Therefore, we emphasize that our results probably cannot be generalized beyond distal metaphyseal forearm fractures.

Remanipulations were performed only in eight cases even though LOR was documented in 25 cases. This difference between hospital guidelines and the reality has been reported previously and may be explained by the confidence of a satisfactory outcome due to the high remodeling potential in DFFs [7].

Several limitations should be noted. First, the remodeling potential of displaced DFFs is high, yet opinions among experts differ about the acceptable degree of angulation. This limits comparability with previous studies, especially those that used other angulation limits. We chose a limit that avoids prolonged remodeling at the time of cast removal and therefore had a very high compliance among patients and parents, since no functional handicap could result from taking part in our study.

Second, we excluded completely displaced DFF because different opinions exist whether such fractures benefit from osteosynthesis or not; this probably influences our LOR rate compared with other studies.

In conclusion, immobilization of reduced DFFs with FG short-arm casting is as effective as with long-arm casting in the treatment of children aged four years and older. When using a SAC, patients had less restriction in ADL and regained normal motion of the elbow faster than children treated with LACs.
